# Management of patients with Cushing’s disease in the Gulf Region: a Delphi consensus recommendation

**DOI:** 10.3389/fendo.2025.1665985

**Published:** 2025-09-30

**Authors:** Mussa H. Almalki, Tarik Elhadd, Khaled M. AlDahmani, Aishah Ekhzaimy, Abdullah Alqanaei, Hasan F. Jamal, Abdulla Alfutaisi, Moeber Mahzari, Salem A. Beshyah, Ali S. Alzahrani

**Affiliations:** ^1^ Obesity, Endocrine, and Metabolism Center, King Fahad Medical City, Second Health Cluster, Riyadh, Saudi Arabia; ^2^ College of Medicine, Alfaisal University, Riyadh, Saudi Arabia; ^3^ Endocrine Section, Department of Medicine, Hamad Medical Corporation, Doha, Qatar; ^4^ Division of Endocrinology, Department of Medicine, Tawam Hospital, Al Ain, United Arab Emirates; ^5^ Department of Internal Medicine, College of Medicine and Health Sciences, United Arab Emirates University, Al Ain, United Arab Emirates; ^6^ Division of Endocrinology, Department of Medicine, Sheikh Tahnoon Bin Mohammed Medical City, Al Ain, United Arab Emirates; ^7^ Endocrinology and Diabetes Unit, Department of Medicine, College of Medicine and King Saud University Medical City, King Saud University, Riyadh, Saudi Arabia; ^8^ Division of Endocrinology, Department of Medicine, Sabah Hospital, Kuwait City, Kuwait; ^9^ Division of Endocrine & Diabetes, Salmanyia Medical Complex, Governmental Hospital, Ministry of Health, Manama, Bahrain; ^10^ Department of Medicine, College of Medicine, Sultan Qaboos University, Muscat, Oman; ^11^ College of Medicine, King Saud bin Abdulaziz University for Health Sciences, Riyadh, Saudi Arabia; ^12^ King Abdullah International Medical Research Center, Riyadh, Saudi Arabia; ^13^ Department of Medicine, Ministry of National Guard Health Affairs, Riyadh, Riyadh, Saudi Arabia; ^14^ Department of Medicine, College of Medicine, Dubai Medical University, Dubai, United Arab Emirates; ^15^ Department of Endocrinology, Bareen International Hospital, Abu Dhabi, United Arab Emirates; ^16^ Department of Molecular Oncology, King Faisal Specialist Hospital & Research Centre, Riyadh, Saudi Arabia; ^17^ Department of Medicine, King Faisal Specialist Hospital & Research Centre, Riyadh, Saudi Arabia

**Keywords:** Cushing disease (CD), Delphi consensus, Gulf region, transphenoid surgery, pasireotide, steroidogenesis inhibitors, radiotherapy, bilateral adrenalectomy

## Abstract

**Introduction:**

Cushing’s disease (CD), most commonly caused by ACTH-secreting pituitary adenomas, is a rare but serious endocrine disorder characterized by chronic hypercortisolism. CD is associated with significant morbidity and increased mortality, necessitating timely and effective intervention.

**Objectives:**

This study aimed to establish consensus-based clinical practice guidelines for managing CD in the Arabian Gulf region, where disparities in healthcare infrastructure and access to therapies present challenges to optimal care delivery.

**Methods:**

A Delphi consensus approach was employed, involving 83 endocrinologists with ≥5 years of independent practice from the six Gulf Cooperation Council (GCC) countries. A scientific committee developed 21 statements covering surgical, medical, and radiotherapeutic management. Consensus was predefined as ≥80% agreement on a 5-point Likert scale.

**Results:**

The Delphi survey revealed strong expert consensus on CD management: nearly all agreed on referral to specialized centers (98.8%) and endorsed transsphenoidal surgery (100%) as first-line treatment. For persistent/recurrent disease, repeat surgery was favored when feasible (91.3%), while medical therapy (e.g., pasireotide or steroidogenesis inhibitors) was preferred for inoperable cases. Drug choice depended on clinical context, with radiotherapy (98.8%) reserved for refractory cases and bilateral adrenalectomy (95.2%) as a last resort. Monitoring protocols, including glycemic and adrenal function assessments, achieved high agreement (97.6–100%).

**Conclusion:**

The study provides structured, region-specific recommendations for CD management in the Gulf region, emphasizing surgical intervention where feasible, tailored medical therapy, and careful monitoring. These guidelines aim to standardize care, address resource limitations, and improve patient outcomes.

## Introduction

Cushing’s disease (CD) is most commonly caused by benign adrenocorticotropic hormone (ACTH)-secreting pituitary adenomas (PAs). It is a rare but serious disorder characterized by chronic hypercortisolism. The estimated incidence of CD ranges from 1.2 to 2.4 per million people per year, and the prevalence is around 57 cases per million (1, 2). However, CD is likely underdiagnosed due to its nonspecific symptomatology and insidious course. CD predominantly affects women, most often manifesting in the third to fifth decades of life (3, 4). It is responsible for about 70% of all cases of endogenous Cushing’s syndrome (CS).

The clinical presentation of Cushing’s disease is variable and frequently includes central obesity, hypertension, glucose intolerance or diabetes mellitus, dyslipidemia, myopathy, osteoporosis, and neuropsychiatric symptoms (4). These comorbidities contribute significantly to an elevated cardiovascular risk and a up to fourfold increase in all-cause mortality (5–7). Therefore, timely diagnosis and coordinated multidisciplinary treatment strategies are crucial for better clinical outcomes and quality of life (8, 9).

The primary objectives of therapy are to restore normal cortisol levels, alleviate clinical manifestations, manage associated comorbidities, inhibit tumor progression, achieve sustained remission, and ultimately reduce disease-related morbidity and mortality. Transsphenoidal surgery (TSS) for PAs remains the first-line treatment for CD (10). However, approximately 20% of patients fail to achieve remission following surgery, and about 15% of those who initially respond experience disease recurrence (11). This means that nearly one-third of CD patients do not achieve long-term cure after pituitary surgery and may require further therapeutic interventions (12). In addition, some patients who are not cured by initial surgery or develop recurrence may not be good candidates for redo-TSS due to clinically significant comorbidities, large tumor size, difficult surgical access, or the patient’s choice. In such cases, alternative approaches such as medical therapy or radiotherapy are considered. In refractory and selected cases, bilateral adrenalectomy could be an option (13).

Medical therapy is a crucial modality in managing CD. It can be used preoperatively, especially in patients with severe CD, to reduce cortisol levels and stabilize the clinical condition prior to surgery (12). Additionally, it can serve as an alternative therapy to surgery in cases of persistent or recurrent disease, or as a bridging option before or after pituitary radiotherapy. Medical therapy may also be the primary treatment of choice, particularly when pituitary surgery is not feasible, or when the patient declines surgical intervention (14). Currently, the pharmacological options for managing CD fall into three major classes: (1) pituitary-directed agents (e.g., cabergoline, pasireotide), which suppress ACTH secretion, (2) adrenal-directed agents or steroidogenesis inhibitors (e.g., ketoconazole, metyrapone, osilodrostat, mitotane, etomidate), and (3) glucocorticoid receptor antagonists (e.g., mifepristone, relacorilant) (15, 16).

In the Arabian Gulf region, the approach to treating CD varies widely due to disparities in healthcare systems and access to available therapies. For example, TSS and radiotherapy are not uniformly available across all countries. In addition, practicing physicians have diverse training background, and many institutions lack a multidisciplinary management approach to CD. These disparities highlight the need for region-specific clinical guidelines on the management of CD. Establishing such guidelines would support standardized treatment approaches, promote uniformity in clinical decision-making, and enable healthcare providers to deliver evidence-based care within the limitations of their local healthcare systems.

To address this need, a region-specific consensus on the management of CD was developed using the Delphi survey methodology (15, 17).

## Methodology

### The Delphi method

Given the limited research on CD, particularly in the Gulf Cooperation Council (GCC) region, this study employed the Delphi method to gather expert consensus and develop regional clinical recommendations (18). The Delphi technique is a structured process in which a panel of experts anonymously provides input on specific, predesigned questions, making it particularly useful for developing clinical guidelines based on experts’ opinions, taking into account available evidence and local resources (19). This study adhered to the DELPHISTAR reporting guidelines for Delphi studies, with the completed checklist available as Supplementary Material.

### Participants

A scientific committee composed of 10 senior endocrinologists with extensive experience in the management of PAs and particularly CD (MA, TE, KA, AE, AA, HF, AF, MM, SB, AA) from all six GCC countries (Saudi Arabia, UAE, Qatar, Oman, Kuwait, and Bahrain) carefully developed the study objectives and Delphi statements. Based on the most pressing need in the region, it was agreed that these guidelines would focus on the management of CD and would not cover diagnostic evaluation or other aspects of CD. After several rounds of meetings and testing of the survey statements by the scientific committee, and upon achieving consensus among members, the survey was distributed to 300 endocrinologists actively managing CD patients across the GCC. Participants were identified through national endocrine societies and professional networks, ensuring representation from university hospitals, government institutions, and private practices to ensure broad representation across GCC countries.

**Figure 1:**
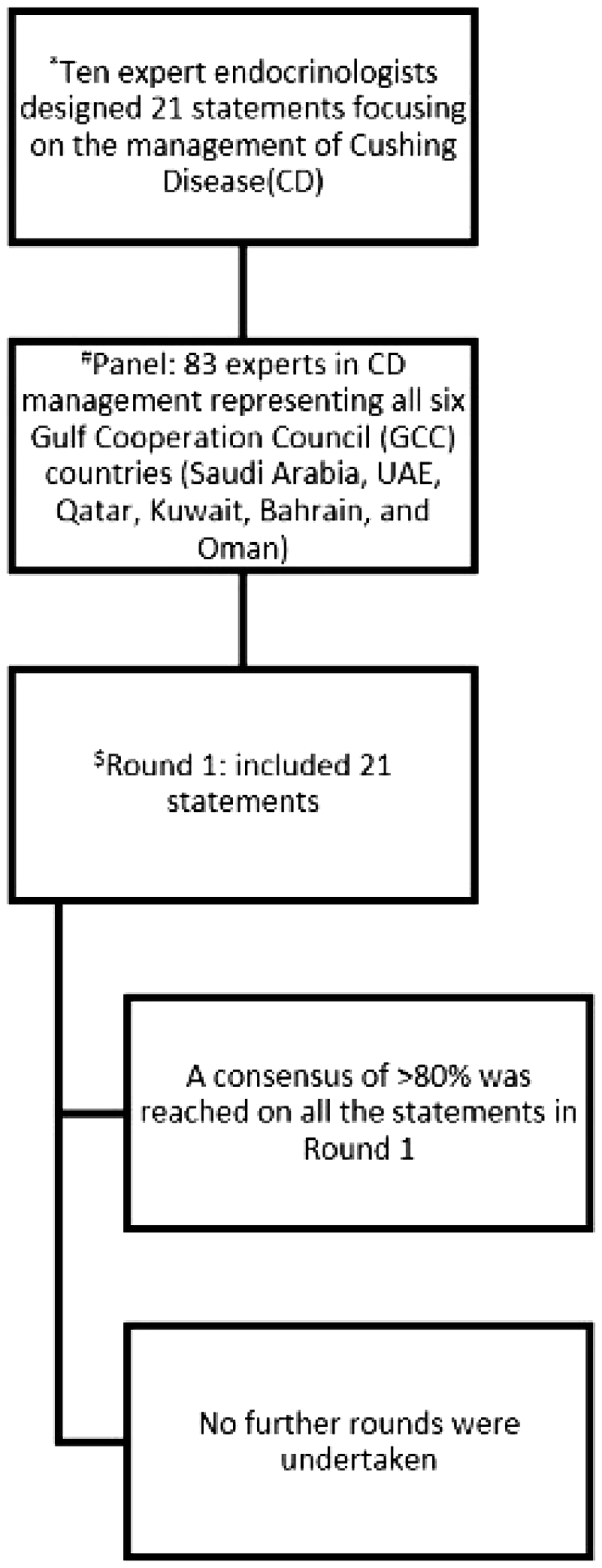
Summary of the process of the Delphi survey to reach consensus. ^*^ The 21 statements developed by the scientific committee based on comprehensive review of the literature, international guidelines, relevant local and international clinical studies, as well as consideration of regional resources. ^#^Three-hundred endocrinologists actively managing CD patients across the GCC were invited, 83 filled the survey; this represents a response rate of 28%. ^$^Consensus was defined as ≥80% agreement.

Eighty-three endocrinologists (response rate 28%) with active CD management experience participated, representing diverse clinical expertise: 65% with >10 years’ experience, 30% with 5–10 years, and 5% with 1–5 years (**Figure 1**).

### The questionnaire

The scientific committee developed the 21 consensus statements through an evidence-based process beginning with a comprehensive literature review of international guidelines and relevant local and international clinical studies, as well as consideration of regional resources available for their availability in the GCC. This review identified key areas of clinical uncertainty and regional practice variation in CD management, which informed the initial statement drafting. Through an iterative refinement process involving two rounds of structured email discussions followed by virtual consensus meeting with all committee members. During these sessions, each statement underwent rigorous evaluation to ensure it addressed clinically relevant uncertainties specific to GCC practice. The finalized statements addressed surgical (n=7), medical (n=9), and radiotherapy (n=5) management. These were incorporated into an anonymous online survey using a 5-point Likert scale, allowing participants to reflect both clinical expertise and local practice contexts. Diagnostic evaluation was intentionally excluded from this consensus as existing international guidelines already provide well-established diagnostic criteria that have been widely adopted across the region. Furthermore, GCC laboratories consistently utilize standardized hormone assay methodologies, reducing local variability in diagnostic interpretation. The committee prioritized addressing identified gaps in therapeutic decision-making specific to regional practice patterns.

### Data analysis

The analysis employed descriptive statistics to evaluate panelists’ responses, with consensus predefined as ≥80% agreement (combining strongly agree and agree responses) (18–20). All 21 statements met this threshold in the first round, eliminating the need for subsequent Delphi rounds (**Figure 1**) with agreement rates ranging from 80.7% to 100%, where higher percentages (e.g., 100%) reflected unanimous agreement and lower percentages (e.g., 80.7-85.8%) indicated more debated topics. The interquartile range (IQR) of agreement rates (84.4–97.6%) indicated that the majority of statements clustered in the high-consensus range, demonstrating strong overall agreement with moderate variability. The response rate was 28% (83/300 invited specialists), consistent with typical Delphi studies, and no attrition occurred as all participants completed the survey.

## Results

The Delphi consensus survey on the management of CD in the Gulf region revealed strong agreement among participants across key aspects of patient care. The proportion of Delphi panelists who indicated some or complete agreement or disagreement with each statement is demonstrated in **Table 1**. A near-unanimous consensus (98.8%) supported referring patients to specialized centers with multidisciplinary expertise, including endocrinology, neurosurgery, radiation oncology, pituitary pathology, and radiology. Surgical intervention with TSS was unanimously recognized (100%) as the primary treatment for patients with confirmed CD due to PA and acceptable surgical risk. In cases of persistent or recurrent disease, repeat TSS was the preferred approach (91.3%) when preoperative imaging identified a resectable tumor, for patients awaiting surgery, or those unsuitable for immediate surgical intervention. Preoperative medical therapy was recommended by a majority (80.8%) for these patients. Adrenal-directed treatments, such as steroidogenesis inhibitors, were favored (92.8%) for rapid control of hypercortisolemia, while long-term medical therapy was strongly supported (98.8%) for patients with inoperable tumors. Pasireotide and steroidogenesis inhibitors (e.g., ketoconazole, metyrapone, osilodrostat) were supported as alternatives, with 88% and 91.6% consensus, respectively. Notably, steroidogenesis inhibitors were preferred over pasireotide in patients with significant hyperglycemia (HbA1c >8%; 85.8%), while pasireotide was favored for those with larger PAs (84.4%). Monitoring protocols were emphasized, with unanimous agreement (100%) on the regular glycemic monitoring of pasireotide-treated patients and a 97.6% consensus on adrenal insufficiency screening for those on steroidogenesis inhibitors. Cabergoline was considered an option for mild, persistent, or recurrent CD (80.7%), and glucocorticoid receptor antagonists (e.g., mifepristone) were suggested for uncontrolled disease with severe metabolic complications (84.3%). For refractory disease, combination therapy targeting multiple mechanisms was widely supported (97.6%), though experts advised caution due to the higher risk of adverse events at increased dosages (96.4%). Radiotherapy was recommended for persistent or recurrent CD when surgery was not feasible (98.8%), especially in resource-limited settings (94%). Bilateral adrenalectomy was reserved for severe, refractory hypercortisolism (95.2%), with 91.5% emphasizing its role as a last-line option due to risks of permanent adrenal insufficiency and Nelson syndrome. Overall, the findings highlight a structured, patient-tailored approach that prioritizes multidisciplinary care, surgical intervention when feasible, and the cautious use of medical and adjunctive therapies, with close monitoring for complications.

**Table 1:** The proportion of Delphi panelists who indicated some or complete agreement/disagreement with each statement.

Statement	Consensus (%)
Patients with Cushing’s disease should be referred to a specialized center with multidisciplinary expertise (endocrinology, neurosurgery, radiation oncology, and radiology).	98.8
Transsphenoidal adenomectomy (TSS) is the first-line therapy for Cushing’s disease in patients with acceptable surgical risk and a confirmed pituitary adenoma.	100
Repeat TSS is the preferred approach for persistent or recurrent Cushing’s disease in patients with acceptable surgical risk and a visible, resectable tumor on MRI.	91.3
Preoperative medical therapy is recommended for all patients with Cushing’s disease if TSS is delayed.	80.8
Adrenal-directed therapies (e.g., steroidogenesis inhibitors) are preferred over pituitary-directed therapies when rapid cortisol normalization is required.	92.8
Long-term medical therapy is recommended for patients with persistent or recurrent Cushing’s disease who are not surgical candidates due to a non-resectable tumor or high surgical risk.	98.8
Treatment with pasireotide should be considered in patients with persistent or recurrent Cushing’s disease who are not surgical candidates due to a non-resectable tumor or high surgical risk.	88
Treatment with steroidogenesis inhibitors (e.g., ketoconazole, metyrapone, osilodrostat) should be considered in patients with persistent or recurrent Cushing’s disease who are not surgical candidates due to a non-resectable tumor or high surgical risk.	91.6
Treatment with steroidogenesis inhibitors (e.g., ketoconazole, metyrapone, osilodrostat) is preferred over pasireotide in patients with active Cushing’s disease and clinically significant hyperglycemia (i.e., HbA1c >8%).	85.8
Treatment with pasireotide is preferred over steroidogenesis inhibitors in patients with active Cushing’s disease and a relatively large pituitary tumor.	84.4
Patients receiving pasireotide should undergo regular glycemic monitoring due to the risk of hyperglycemia.	100
Regular monitoring for adrenal insufficiency is recommended in patients treated with steroidogenesis inhibitors (e.g., ketoconazole, metyrapone, osilodrostat).	97.6
Treatment with cabergoline may be considered in patients with mild persistent/recurrent Cushing’s disease who are not surgical candidates due to a non-resectable tumor or high surgical risk.	80.7
Preoperative or long-term treatment with a glucocorticoid receptor antagonist (e.g., mifepristone) is an option in patients with uncontrolled Cushing’s disease, particularly those with severe metabolic complications (e.g., diabetes mellitus).	84.3
In patients with uncontrolled Cushing’s disease, combination therapy with agents of different mechanisms of action (e.g., pasireotide, cabergoline, osilodrostat, ketoconazole, metyrapone) should be considered.	97.6
The risk of adverse effects should be carefully evaluated and monitored, especially when selecting combination therapies.	98.8
The risk of adverse effects increases with higher drug dosages.	96.4
Radiotherapy should be considered in patients with persistent or recurrent Cushing’s disease and residual pituitary adenoma who are not surgical candidates due to a non-resectable tumor or high surgical risk and have failed medical treatment.	98.8
Radiotherapy should be considered in patients with persistently active Cushing’s disease who are not surgical candidates due to a non-resectable tumor or high surgical risk when medical therapy is unavailable or unaffordable.	94
Bilateral adrenalectomy may be considered in patients with severe hypercortisolism unresponsive to other treatments.	95.2
Bilateral adrenalectomy should be reserved as the last treatment option for Cushing’s disease, as it has important long-term implications, such as permanent adrenal insufficiency and the potential development of Nelson syndrome.	91.5

## Discussion

CD remains a significant clinical challenge globally, with complexity in regions with variable healthcare resources, such as the GCC countries. This Delphi consensus survey involving 83 endocrinology experts from the region establishes a practical, regionally adapted framework for managing CD, aligned with international best practices, while addressing local needs. Successful implementation will require tailored approaches: tertiary centers should establish multidisciplinary CD clinics with standardized protocols, while community hospitals can focus on developing referral pathways and basic management competencies through telemedicine collaborations. All institutions should participate in regional registries to track outcomes, with phased implementation beginning at academic centers before system-wide rollout. Particular attention should be given to equitable access to novel therapies and specialized surgical expertise across urban and rural settings.

Nearly all panelists (98.8%) agreed that CD patients should be referred to specialized centers with multidisciplinary expertise, reinforcing global recommendations that emphasize the importance of coordinated care in improving outcomes and reducing morbidity and mortality associated with hypercortisolism (10, 21). Multidisciplinary management is crucial given the heterogeneous and often subtle presentation of CD, which frequently leads to delayed diagnosis and underrecognized disease burden (10, 21, 22).

TSS remains the first-line treatment for CD in patients with confirmed PAs and acceptable surgical risk, as unanimously supported (100%) by panelists and major international guidelines (10, 22, 23). While initial remission rates after TSS can reach 70–85% for microadenomas, recurrence rates remain substantial (20–30%), highlighting the importance of long-term follow-up and patient counselling (10, 12, 24–27). For patients with persistent or recurrent disease, repeat TSS is the preferred approach (91.3% consensus) when a resectable tumor is identified, consistent with international practices (10, 24). However, a significant proportion of patients are not candidates for surgery due to comorbidities, tumor location, or patient preference, necessitating alternative therapeutic strategies (27).

Medical therapy is an essential component of CD management, both as a preoperative measure to stabilize severe hypercortisolism and as a long-term treatment for non-surgical candidates or those with persistent disease (4, 27). Most experts (80.8%) endorsed preoperative medical therapy when TSS was delayed, and nearly all (98.8%) supported long-term medical therapy for patients who were inoperable.

Adrenal steroidogenesis inhibitors (e.g., ketoconazole, metyrapone, osilodrostat) were preferred for rapid cortisol control (92.8%), especially in the presence of significant hyperglycemia, given the diabetogenic effects of the primary pituitary directed agents, such as pasireotide (4, 27). However, pasireotide was favored for patients with large PAs (84.4%), due to its potential for tumor shrinkage (27).

The Delphi panel also emphasized the importance of regular monitoring for adverse effects with 100% consensus for glycemic monitoring with pasireotide and 97.6% for adrenal insufficiency with steroidogenesis inhibitors, aligning with the increasing recognition of the need for proactive management of therapy-related complications, which can significantly impact patient outcomes and quality of life (10, 27).

Our proposed monitoring approach reflects established strategies in the literature. For pasireotide therapy, baseline assessment of fasting plasma glucose (FPG) and glycated hemoglobin (HbA1c) is essential, with weekly glucose monitoring recommended during the first three months of treatment and for 4–6 weeks following dose adjustments (28, 29). Long-term monitoring should include periodic FPG and HbA1c measurements, with consideration of continuous glucose monitoring for high-risk patients; incretin-based agents (GLP-1 receptor agonists or DPP-4 inhibitors) are preferred for managing treatment-emergent hyperglycemia (30). For osilodrostat therapy, regular monitoring of morning serum cortisol and urinary free cortisol (every 1–2 weeks during titration, then every 1–2 months during maintenance) is critical to detect adrenal insufficiency, with glucocorticoid replacement initiated for cortisol levels <5 μg/dL or clinical symptoms (31, 32). Monitoring should continue after treatment discontinuation until biochemical normalization is confirmed. These protocols emphasize proactive surveillance to optimize the safety profile of these targeted therapies (33).

Cabergoline was considered for mild, persistent, or recurrent CD (80.7%), consistent with its established though modest efficacy (27). Glucocorticoid receptor antagonists (e.g., mifepristone, relacorilant) were recommended for uncontrolled disease with severe metabolic complications (84.3%), supported by recent evidence of their benefits in improving glycemic control (10, 27). Combination therapy targeting multiple mechanisms of action was widely endorsed (97.6%), though experts cautioned about a higher risk of adverse effects at increased dosages (98.8%) (27).

Radiotherapy remains an important option for patients with persistent or recurrent CD who are not surgical candidates (98.8%), particularly in resource-limited settings (94%). While effective, radiotherapy is associated with delayed therapeutic effects and an increased risk of hypopituitarism, necessitating close endocrine follow-up (24, 27). Bilateral adrenalectomy was reserved for severe, refractory hypercortisolism (95.2%), with 91.5% of panelists emphasizing its role as a last resort due to the risk of permanent adrenal insufficiency and Nelson syndrome (occurring in up to 25% of cases) (10, 24).

Despite advances in therapy, CD patients continue to experience excess morbidity and mortality, particularly from cardiovascular disease, infections, and thromboembolic events, even after biochemical remission (1, 4). Neurocognitive impairment and mental fatigue are also common, highlighting the need for lifelong multidisciplinary follow-up (22).

This study highlights the specific challenges faced in the Gulf region, including disparities in access to specialized care, advanced surgical techniques, and novel medical therapies (27). Regionally adapted consensus guidelines, as presented here, are crucial for standardizing care, enhancing early detection, and ensuring equitable treatment. An earlier questionnaire-based survey on management of Cushing disease spanned a wider geographical area of the Middle East and North Africa with a more variable economic status (27). The current study focused on the Gulf States with more homogenous access to care resources and therefore its results may be more applicable.

## Limitations and future directions

While these consensus recommendations represent an important step toward standardizing CD management in the Gulf region, several limitations must be acknowledged. First, this Delphi process did not incorporate direct patient or public perspectives, which could have provided valuable insights into treatment preferences, quality-of-life priorities, and practical barriers to care adherence. Second, the reliance on expert opinion through consensus methods may introduce bias and may not fully reflect the diversity of clinical practices across all GCC countries. Third, the absence of robust local epidemiological and outcome data significantly limits the generalizability of these recommendations, highlighting the need for future multicenter studies to address this critical gap. Finally, the considerable variability in healthcare infrastructure and access to advanced therapies across different GCC nations may pose challenges to uniform implementation of these guidelines. Future directions should focus on integrating newer therapies (e.g., osilodrostat, levoketoconazole, and relacorilant) (27) into regional practice and advancing research into the molecular pathogenesis of CD to develop more targeted treatments. Technological advances in imaging, biomarkers, and minimally invasive surgical techniques may further enhance early diagnosis and treatment efficacy (27). However, realizing these advances will require substantial investment in healthcare infrastructure and specialized training programs, particularly in more resource-limited settings within the region. Implementation of these recommendations will require thoughtful local adaptation and validation through real-world clinical studies to assess their effectiveness across different practice environments.

## Conclusion

This Delphi consensus provides a practical, regionally tailored framework for managing CD in the Arabian Gulf, emphasizing multidisciplinary care, individualized therapy, and vigilant long-term follow-up. Adoption of these evidence-informed recommendations is expected to improve clinical outcomes, reduce treatment variability, and support equitable healthcare delivery across the region.

## Data Availability

The original contributions presented in the study are included in the article/**Supplementary Material**. Further inquiries can be directed to the corresponding author.

## The Gulf Pituitary Consensus Group

Mussa H Almalki, Tarik Elhadd, Khaled AlDahmani, Aishah Ekhzaimy, Abdullah Alqanaei, Hasan F. Jamal, Abdulla Alfutaisi, Moeber Mahzari, Salem A. Beshyah, Ali S. Alzahrani, Hassan Shawa, Bandar Damanhori, Awad Alshahrani, Bashir Taha Salih, Abbas Ali Mansour, Abdulraof Almahfouz, Najat Al Harthi, Omar Dhaimat, Adel Saleh Alghamdi, Turki Alharthi, Khaled Baagar, Ali Hilal Al Maqbali, Abdulkareem Almalki, Yousef Alansari, Majed Al Adwani, Noha N. Mukhtar, Ashwag Alqahtani, Ahmed Abdul Karim Hassoun, Ahmad Esmaeel Abdullah, Husain Taha, Jehan Abdulla, Ahmad AlKandari, Gubartallah Abdalmunaim Khogli, Khalid Hassan, Amin Jayyousi, Omar Alhuzaim, Ahmed Sadiq Al Sajwani, Salim Al Qassabi, Arwa Alyamani, Wiam Hussein, Khadija Hafidh, Maram AlSubaiee, Ibrahim Alsadhan, Ahmad Saad Al Ghamdi, Ali Al Reesi, Nawal Alotaibi, Imad Brema, Ahmad M. Al Furqani, Abdullah Nasser AlMuqawed, Ohoud Almohareb, Sara Gaafar Ibnauf Suliman, Abdullah A. Alamri, Fatima Bahowairath, Ahmad Sumaily, Muneera Hadi Al-Bogami, Hazem Aljumah, Sadiq Juma Al lawati, Muhammad Mujammami, Zaina Rohani, Jenan Ebrahim Obaid, Saleha Babli, Reem Alamoudi, Mohammed S. Almohaya, Eman Alfadhli, Hamzah Khalid Sab, Waleed Mater Al Mutairi, Naji Aljohani, Sameerah Alshehri, Nasser Alqattan, Abdulrahman Almaghamsi, Amani Alhozali, Lolwah Alashgar, Wael Mohammad Almistehi, Nasreen Alsayed, Jim Philip, Ahmad Alhashemi, Waleed Saleh Almutairi, Saad H. Alzahrani, Raed Alenezi, Mohammed Bashir, Dalal Alromaihi, Magdi Eid Nasr Elesseily, Saud Al Harthi.
