# Supplementary material for: Management of patients with Cushing’s disease in the Gulf Region: a Delphi consensus recommendation
Source: Front Endocrinol (Lausanne). 2025 Sep 30;16:1665985. doi: 10.3389/fendo.2025.1665985 (PMC12518058; doi:10.3389/fendo.2025.1665985)
Supplement: Supplementary file 1 [file Table1.docx]

**S1 File: Delphi studies in social and health sciences – recommendations for an interdisciplinary standardized reporting (DELPHISTAR).**

**This reporting guideline is meant for studies using Delphi techniques in the health and social sciences.**

| **Topic** | **Section** | **Item** | **Checklist Item** | **Location where item is reported** | **Exemplary answer** |
| --- | --- | --- | --- | --- | --- |
| **I**  **Title and Abstract** |  | 1 | Identification as a Delphi procedure in the title | Title | Results of a Delphi study. |
|  |  | 2 | Identification as a Delphi procedure in the abstract | Abstract | A Delphi procedure was selected to answer the research question. |
|  |  | 3 | Structured abstract | Abstract | e.g., background, method, results and discussion |
| **II**  **Context** | **Formal** | 4 | Information about the sources of funding | Disclosures | No funding |
|  |  | 5 | Information about the team of authors and/or researchers (e.g., discipline, institution) | Methodology | The Delphi study was conducted by an interdisciplinary team with representatives from Endocrinology in Gulf Cooperation Council (GCC) region. |
|  |  | 6 | Information about method consulting | Methodology | No outside consulting in regard to method took place. |
|  |  | 7 | Information about the project background | Introduction and Methodology | The Delphi survey utilized to develop the consensus |
|  |  | 8 | Information about the study protocol | Methodology | The study protocol is detailed in the methodology section |
|  | **Content** | 9 | Justification of the chosen method (Delphi procedure) to answer the research question | Methodology | The Delphi method is suitable for answering the research question because it systematically gathers the judgments of different experts and can identity agreement and disagreement. |
|  |  | 10 | Aim of the Delphi procedure (e.g., consensus, forecasting) | Introduction and Methodology | The aim of the Delphi study is to find consensus on management of Cushing disease(CD) |
| **III**  **Method** | **Body & Integration of knowledge** | 11 | Identification and elucidation of relevant expertise, spheres of experience, and perspectives (e.g., theory, practice, affected groups, disciplines) | Methodology | The experts represented the spectrum of endocrinologists in the GCC region |
|  |  | 12 | Handling of knowledge, expertise and perspectives which are missing or have been deliberately not integrated | Not applicable | Experts were recruited for this study |
|  |  | 13 | Basic definition of expert^1^ | Methodology | A person who has been active in the area for at least >1 year managing patients with CD is considered to be an expert. |
|  | **Delphi variations** | 14 | Identification of the type of Delphi procedure and potential modifications (e.g., classic Delphi, real-time Delphi, group Delphi) | Methodology | A classic Delphi procedure was used. |
|  |  | 15 | Justification of the Delphi variation and modifications, including during the Delphi process, if applicable | Methodology and Results | The consensus of >80% was reached on all the statements from round 1, therefore no other rounds were undertaken |
|  | **Sample of experts** | 16 | Selection criteria for the experts (per round if there are different expert groups) | Methodology | All of the experts who met the definition were invited to the first round.  No subsequent rounds. |
|  |  | 17 | Identification of the experts | Methodology | The experts were identified based on their clinical practice |
|  |  | 18 | Information about recruiting and any subsequent recruiting of experts | Methodology | The experts were informed about the Delphi study and invited to participate. |
|  | **Survey** | 19 | Elucidation of the content development for the questionnaire^2^ | Methodology | The questionnaire was developed by a panel of scientific committee based on the results of literature review. |
|  |  | 20 | Description of the questionnaire (content and structure) | Methodology | The questionnaire was focused on the management of CD. The statements made in the questionnaire were evaluated using standardized items |
|  | **Delphi rounds** | 21 | Number of Delphi rounds | Methodology and Result | One Delphi round was held and consensus of >80% was achieved for all the statements in the first round |
|  |  | 22 | Information about the aims of the individual Delphi rounds | Methodology and Result | The first and only Delphi round was focused on the management of CD |
|  |  | 23 | Disclosure and justification of the criterion for discontinuation | Methodology | The number of rounds was defined in advance to be a maximum of three rounds. However, since the consensus on all the statement was reached in the first round no further rounder were undertaken. |
|  | **Feedback** | 24 | Information about what data was reported back per round | Methodology and Result | In terms of feedback, we shared the statistical results. |
|  |  | 25 | Information on how the results of the previous Delphi round were fed back to the experts surveyed (e.g., via frequencies, mean values, measures of dispersion, listing of comments) | Methodology and Result | Mean values, standard deviations and percentage frequency distributions were reported. |
|  |  | 26 | Information on whether feedback was differentiated by specific groups (e.g., by field of expertise, institutional affiliation) | Methodology and Result | The feedback was aggregated across all expert groups. |
|  |  | 27 | Information about how dissent and unclear results were handled | Not applicable | Not applicable |
|  | **Data analysis** | 28 | Disclosure of the quantitative and qualitative analytical strategy | Methodology and Result | The quantitative items were descriptively analyzed. |
|  |  | 29 | Definition and measurement of consensus | Methodology and Result | Consensus was defined as percentage agreement, meaning that agreement was assumed if at least 80% of the respondents agreed on an item. |
|  |  | 30 | Information on group-specific analysis or weighting of experts (e.g., theory vs. practice, discipline-specific analysis) | Not applicable | Not applicable |
| **IV**  **Results** | **Delphi process** | 31 | Illustration of the Delphi process (e.g., in a flow chart) | Figure 1 | A summary of the process is illustrated in a flow chart (Figure 1). |
|  |  | 32 | Information about special aspects during the Delphi process (e.g., deviations from the intended approach with justification) | Methodology and Result | During the Delphi procedures the consensus was reached on all the statements in round 1, therefore no further rounds were undertaken |
|  |  | 33 | Number of experts per round (both invited and participating) | Methodology | The number of experts participating in the first and only Delphi round was 83, This corresponds to a response rate of 83% |
|  | **Results** | 34 | Presentation of the results for each Delphi round and the final results | Result | In the first and only Delphi round >80% of the respondents agreed on all the statements |
| **V Discussion** | **Quality of findings** | 35 | Highlighting the findings from the Delphi study | Result and Discussion | The study finding include consensus on the management of CD emphasizing the importance of surgical and medical treatment options. |
|  |  | 36 | Validity of the results (e.g., transferability of the findings) | Result and Discussion | The results are consistent with other international guidelines on the management of CD. |
|  |  | 37 | Reliability of the results (e.g., split half, inter-rater reliability) | Not applicable | Not applicable |
|  |  | 38 | Reflection on potential limitations (e.g., distortion, skewing, bias) | Discussion | The results are to be viewed critically with regard to the composition of the panel because all practicing the GCC region. |

^1^ “Experts” are the participants; this can be people from academia, practice, or representatives of lived experience (e.g., patients, family members).

^2^ The term “questionnaire” stands for the survey instrument regardless of whether quantitative or qualitative items are integrated or weighted.
